# Reliability of streetscape audits comparing on‐street and online observations: MAPS-Global in 5 countries

**DOI:** 10.1186/s12942-021-00261-5

**Published:** 2021-01-28

**Authors:** Ana Queralt, Javier Molina-García, Marta Terrón-Pérez, Ester Cerin, Anthony Barnett, Anna Timperio, Jenny Veitch, Rodrigo Reis, Alexandre Augusto Paula Silva, Ariane Ghekiere, Delfien Van Dyck, Terry L. Conway, Kelli L. Cain, Carrie M. Geremia, James F. Sallis

**Affiliations:** 1 Department of Nursing, University of Valencia, Jaume Roig s/n, 46010 Valencia, Spain; 2 AFIPS Research Group, University of Valencia, Valencia, Spain; 3 Department of Teaching of Musical, Visual and Corporal Expression, University of Valencia, Valencia, Spain; 4 Mary Mackillop Institute for Health Research, Australian Catholic University, Melbourne, Australia; 5 School of Public Health, The University of Hong Kong, Hong Kong, China; 6 Institute for Physical Activity and Nutrition (IPAN), School of Exercise and Nutrition Sciences, Deakin University, Geelong, Australia; 7 Prevention Research Center, Brown School, Washington University in St. Louis, St. Louis, USA; 8 Research Group on Physical Activity and Quality of Life, Pontificia Universidade Catolica do Parana, Curitiba, Brazil; 9 Graduate Program in Health Sciences, Pontifícia Universidade Católica do Parana, Curitiba, Brazil; 10 Department of Public Health, Ghent University, Ghent, Belgium; 11 Department of Human Biometry and Biomechanics, Vrije Universiteit Brussel, Brussels, Belgium; 12 Research Foundation Flanders (FWO), Brussels, Belgium; 13 Department of Movement and Sports Sciences, Ghent University, Ghent, Belgium; 14 Department of Family Medicine and Public Health, University of California San Diego, San Diego, CA USA

**Keywords:** Built environment, Measurement, Physical activity, Direct observation, Neighborhood

## Abstract

**Background:**

Microscale environmental features are usually evaluated using direct on-street observations. This study assessed inter-rater reliability of the Microscale Audit of Pedestrian Streetscapes, Global version (MAPS-Global), in an international context, comparing on-street with more efficient online observation methods in five countries with varying levels of walkability.

**Methods:**

Data were collected along likely walking routes of study participants, from residential starting points toward commercial clusters in Melbourne (Australia), Ghent (Belgium), Curitiba (Brazil), Hong Kong (China), and Valencia (Spain). In-person on the street and online using Google Street View audits were carried out by two independent trained raters in each city. The final sample included 349 routes, 1228 street segments, 799 crossings, and 16 cul-de-sacs. Inter-rater reliability analyses were performed using Kappa statistics or Intraclass Correlation Coefficients (ICC).

**Results:**

Overall mean assessment times were the same for on-street and online evaluations (22 ± 12 min). Only a few subscales had Kappa or ICC values < 0.70, with aesthetic and social environment variables having the lowest overall reliability values, though still in the “good to excellent” category. Overall scores for each section (route, segment, crossing) showed good to excellent reliability (ICCs: 0.813, 0.929 and 0.885, respectively), and the MAPS-Global grand score had excellent reliability (ICC: 0.861) between the two methods.

**Conclusions:**

MAPS-Global is a feasible and reliable instrument that can be used both on-street and online to analyze microscale environmental characteristics in diverse international urban settings.

## Background

According to ecological models of health behaviors, physical activity (PA) has multiple levels of influence, including built environment characteristics [1]. Environmental factors can be classified as *macroscale* or *microscale* variables [2]. Macroscale attributes are structural features of the environment such as residential density, street connectivity and land use mix that can affect walking to destinations [3]. Microscale characteristics refer to details of streetscapes that can affect the experience of being active, such as design and amenities of streets, sidewalks, and crosswalks, or indicators of social environments and aesthetics [4]. A small body of literature has established strong relationships across age groups between microscale attributes and PA, mainly active transport to destinations, independent of macro-level walkability [2, 5, 6].

Observation, or audit, measures have been developed to evaluate different types of built environments (e.g., urban centers, residential neighborhoods, public open spaces) [7–10]. Studies initially established the inter-rater reliability of these instruments using in-person, on-street measurements [7, 11, 12]. One such instrument is the Microscale Audit of Pedestrian Streetscapes (MAPS) whose items and subscales mainly had moderate to excellent inter-observer reliability [12] and demonstrated validity through associations with several PA measures in multiple age groups [2].

On-street observations usually consume more time and expense than measurements conducted remotely using online imagery, for example Google Street View. Remote online observations reduce travel costs and are particularly useful when evaluating geographically dispersed or international locations [7, 10, 13]. Several studies documented generally strong agreement between on-street and online observations in the USA, Australia and New Zealand [7–9, 14]. For example, Wilson et al. [7] reported significant associations between on-street and Google Street View measures for most items in an instrument applied in two US cities. A shorter version of MAPS (i.e. MAPS Abbreviated Online) was shown to be a reliable online audit tool when compared to on-street assessments [15, 16].

The MAPS-Global observation instrument was based in part on the original MAPS [2] and designed to be appropriate for international use, providing measures of microscale features that are comparable across countries by drawing on items developed across several continents [17]. Because MAPS-Global is the first audit instrument designed for international use, it is important to evaluate its performance across countries with a range of built environment and cultural characteristics. The present study aimed to assess cross-method reliability of MAPS-Global on an international basis by comparing on-street and online observations in five diverse countries.

## Methods

### Microscale Audit of Pedestrian Streetscapes-Global Version (MAPS-Global)

As described elsewhere [17], MAPS-Global was based on the original MAPS tool developed and validated in the US [2, 12]. MAPS-Global was modified substantially by drawing on items from built environment instruments developed on multiple continents: MAPS (US) [12], Bikeability Toolkit (Australia) [18], SPACES (Australia) [19], ALPHA (Europe) [20], REAT (UK) [21], FASTVIEW (UK) [22], school audit tool used in SPEEDY/ ISCOLE study (UK/International) [23], EAST_HK (Hong Kong) [11], NEWS-Africa [24], and NEWS-India [25]. Wording and scoring were altered for greater international applicability and consistency within MAPS-Global. Numerous international investigators provided input and pre-tested drafts [17]. A key purpose was to represent PA-relevant streetscape characteristics that are relevant across diverse geographic settings. If important attributes only seemed relevant in a few locations, they were retained. Thus, MAPS-Global was designed to be tailored to most settings with specialized items, but it also was comparable across countries due to the common instrument. Examples of items common in a subset of settings would include pedestrian streets that are common in Europe but rare in the US, unpaved roads that were common in Africa and India, and cul-de-sacs that are common in the US but not elsewhere [17].

MAPS-Global has 123 items in four sections: overall route, street segments, street crossings, and cul-de-sacs. The route section has three subsections: destinations and land use, streetscape, and aesthetics and social environment. Route items assess characteristics along a short route from a residential starting-point address towards a pre-selected cluster of non-residential land-use destinations (e.g., shopping areas, restaurants). Route items evaluate, for example, presence of non-residential destinations within the short route, aesthetics characteristics, and transit stops. Street segment (defined as the area between street crossings) items measure aspects of sidewalks, bicycle facilities, and pedestrian shortcuts. Crossings items analyze pedestrian protection features and width of crossings. The cul-de-sac section includes size and presence of amenities. The MAPS-Global audit instrument, manual, and training webinars can be found at https://drjimsallis.org/measure_maps.html. MAPS-Global was found to have good inter-rater reliability for on-street observations in 5 countries [17].

### Study design and cities

The present study was conducted in five cities: Melbourne (Australia), Ghent (Belgium), Curitiba (Brazil), Hong Kong (China), and Valencia (Spain). Table 1 indicates study locations and summarizes sample sizes for the MAPS-Global evaluation in each country. This study was developed within the framework of the IPEN (International Physical Activity and the Environment Network) Adolescent project (www.ipenproject.org), which had the goal to represent all inhabited continents with the maximum variability in built environments. Cities included in the present reliability study covered diverse contexts from different continents. For instance, Melbourne represented a low population density city, Curitiba a middle-income site, and Hong Kong a high population density and high-income place [26].

**Table 1:** Study locations, sample sizes and assessment times for MAPS-Global evaluation

Country	City		Sample size								Assessment time (min/route) Mean ± SD (range)^a^	
			Routes	Segments		Crossings		Cul-de-sacs			On-street	Online
Australia	Melbourne		65	208		91		10			19 ± 7 (5–40)	21 ± 7 (9–45)
Belgium	Ghent		81	236		156		6			30 ± 14 (4–85)	26 ± 17 (4–78)
Brazil	Curitiba		82	319		213		0			9 ± 3 (1–23)	11 ± 4 (2–29)
China	Hong Kong SAR		40	115		73		0			17 ± 10 (4–70)	–^b^
Spain	Valencia		81	350		266		0			28 ± 9 (10–45)	32 ± 11 (8–62)
Overall			349	1228		799		16			22 ± 12 (1–85)^c^	22 ± 12 (2–78)

*SD* standard deviation

^a^The remarkably different assessment times by country are due to the characteristics of the different regions evaluated

^b^Start and end assessment times were not collected in Hong Kong for online assessments

^c^This overall value does not include Hong Kong assessment time due to missing data for the online assessment time comparison

Target locations were selected in each city using a geographically stratified sampling design to ensure representation of neighborhoods varying in walkability and socio-economic status (SES). To select high- versus low-walkable neighborhoods, all cities used a GIS-derived macro-level walkability index based on net residential density, intersection density, and mixed land use [27, 28]. High and low SES categories were established using census data about household income or education. Deciles were calculated. The lowest five deciles constituted the “low” category and the highest five deciles corresponded with the “high” category in most cities, while more stringent criteria were applied in Curitiba which excluded the highest, lowest and middle deciles of SES scores [26]. As in previous research [27, 28], a 2 × 2 matrix was defined by high/low walkability and high/low SES. Participants were recruited from areas that met walkability and SES criteria. For the present study, participant addresses were randomly selected and stratified by quadrant, except for Melbourne where general residential addresses were randomly selected from areas within the 2 × 2 matrix. These addresses served as route starting points. Apart from these residence-based routes, to ensure wide variation of contexts, audits were also conducted on segments near some routes which mainly contained retail destinations. These are referred to as commercial routes. IPEN Adolescent was approved for research with human subjects by the Institutional Review Boards at the authors’ universities. Present analyses did not use IPEN Adolescent participant data.

### Data collection

MAPS-Global data were collected on-street and online in 2015 by two independent raters in each country to evaluate cross-method reliability. One rater carried out the observations by walking on-street. The other rater, who was also in-country, carried out online audits, using Google Earth and Google Street View imagery.

Following previous research [2, 12], MAPS-Global observations were conducted along a 400–725 m route from a starting point toward a pre-determined commercial cluster along the street network, to represent a likely walking route. The final sample included 349 routes, 1228 street segments, 799 crossings, and 16 cul-de-sacs (see Table 1). Commercial routes represented approximately 20 % of the final sample.

As mentioned elsewhere [17], a research staff manager from the IPEN Coordinating Center was responsible for training and quality control. Raters were trained in two stages. First, remote training was given to each country’s investigative team by the IPEN coordinating center via a webinar and were provided training materials including a manual with item definitions and photos. Country teams then conducted their own on-street training sessions, sending photos to the coordinating center for clarification. Second, raters were certified by completing at least 5 routes, including at least 2 commercial routes, 5 segments, 5 crossings, and 2 cul-de-sacs/dead ends. When 95 % inter-rater agreement was reached with the trainer at the coordinating center, raters were certified to rate independently. Most raters reached certification during the first round of 5 routes, whilst some required two rounds to reach certification. Investigators were encouraged to hold weekly rater meetings to review questions and concerns, and to minimize rater drift over time.

### Scoring and data analysis

MAPS-Global scoring was similar to that of the original MAPS [12]. Items used a variety of response formats; therefore, all items (except for land uses) were dichotomized or trichotomized to provide relatively equal weighting when creating scales. Land use items were scored as 0, 1, 2, 3, 4 or 5+. Subscales were computed by summing related items after they were rescored. The cul-de-sac section was not analyzed due to the small sample size and unclear expected association with PA. Positive and negative valence scores were created by summing subscales based on expected associations with PA. To create “overall” section scores, negative valence scores were subtracted from positive valence scores. Finally, a grand score was calculated by subtracting the overall negative valence score from the overall positive valence score. Three new conceptual subscales were developed for MAPS-Global, drawing from multiple sections: pedestrian infrastructure, pedestrian design, and bicycle facilities [17]. Detailed information about item recodes and subscale creation can be downloaded (https://drjimsallis.org/Documents/Measures_documents/MAPS%20DATA%20DICTIONARY_GLOBAL_090617.pdf).

### Analyses

Inter-rater reliability analyses were performed using the Kappa statistic for dichotomous variables and Intraclass Correlation Coefficients (ICCs) for continuous or ordinal variables using the one-way random model for average measures, considering values ≥ 0.60 as “good to excellent” reliability, values between 0.41 and 0.60 as “moderate” reliability and values ≤ 0.40 as “fair to poor” reliability [29]. Items rarely observed and with low variability in scores (i.e., almost all zeros or ‘never’) but percentage agreement between raters ≥ 75 % were considered to have good reliability irrespective of low ICCs [19].

Analyses were performed using SPSS version 22 (SPSS Inc., Chicago, IL). For each item (both original and recoded), range, frequency and inter-rater reliability were calculated as well as mean and standard deviations for both on-street and online raters.

## Results

**Fig. 1:**
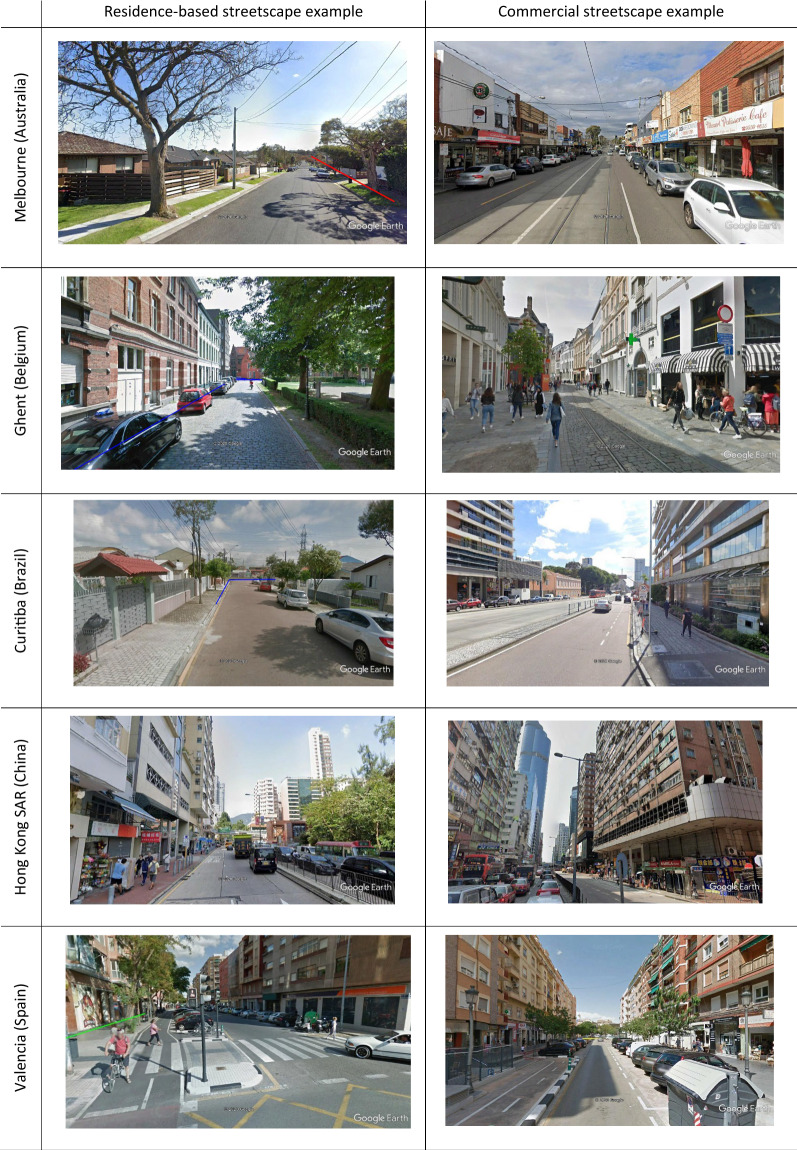
Examples of residence-based and commercial streetscapes for each of the cities

Figure 1 shows images of a sample residential segment and commercial segment for each of the cities. The number of routes, segments and crossings and average assessment times varied across countries (Table 1). With the exception of Belgium, online mean assessment times, not including travel, were a little higher than on-street times. However, overall, mean (± SD) assessment time was 22 ± 12 min for both on-street and online route evaluation.

Table 2 provides route subscale reliability and descriptive analyses. For the destinations and land use subsection, all subscales showed good to excellent reliability between on-street and online raters, with ICCs ranging from 0.680 to 0.859, including the overall score with an ICC value of 0.856. Items that were thought to positively influence walking in the streetscape subsection (such as street amenities and traffic calming signage) were aggregated into a positive valence score, which showed good to excellent reliability (ICC: 0.742). Aesthetics and social subsection subscales also showed good to excellent reliability, including the overall score (ICC: 0.736).

**Table 2:** MAPS-Global Route section reliability scores (n = 349) between on-street and online raters

Subscale	Content	Range of scores	Number of items	Inter-Rater Agreement Kappa/ICC	Rater’s mean (SD)	
					On-street	Online
*Destination and Land Use section (DLU)*						
Residential Mix	1 = Apartment over retail only 2 = Apartments or multi-family only 3 = Mixed or other 4 = Single family only 0 = None	0–4	4	0.823 ICC	2.71 (0.97)	2.76 (0.99)
Commercial-Shops	Grocery, convenience store, bakery, drugstore, other retail, shopping mall, strip mall, open air market	0–26	8	0.838 ICC	4.34 (5.12)	3.76 (4.46)
Commercial -Restaurants/Entertainment	Fast food, sit-down, café, entertainment	0–15	4	0.775 ICC	2.32 (3.21)	1.93 (2.72)
Institutional/Services	Bank, health-related professional, other service	0–15	3	0.859 ICC	4.13 (3.97)	3.47 (3.68)
Public Recreation Facilities	Public indoor, public outdoor pay-to-use, public park and trail	0–5	4	0.752 ICC	0.53 (0.81)	0.51 (0.82)
Private Recreation Facilities	Private indoor and outdoor	0–3	2	0.680 ICC	0.17 (0.48)	0.17 (0.49)
Institutional - Place of Worship	Temple, church, synagogue, convent, mosque, etc.	0–5	1	0.803 ICC	0.25 (0.56)	0.20 (0.49)
Institutional - School	Any type of school	0–5	1	0.692 ICC	0.64 (1.08)	0.44 (0.74)
Pedestrian Street	Pedestrian street or zone	0.5	1	0.902 ICC	0.30 (0.82)	0.33 (0.86)
Overall DLU Negative	Age-restricted bar, liquor or alcohol store	0–6	2	0.696 ICC	0.30 (0.84)	0.34 (0.92)
Overall DLU Positive	Residential Density Mix + Commercial-Shops + Commercial Restaurants/Entertainment + Institutional/Services + Institutional-Place of worship + Institutional-School + Public Recreation Facilities + Private Recreation Facilities + Pedestrian Street or Zone	1–59	28	0.854 ICC	14.62 (12.81)	12.74 (11.36)
Overall DLU Score	DLU Overall Positive Subscale – DLU Overall Negative Subscale	-3-59	30	0.856 ICC	14.32 (12.69)	12.40 (11.35)
*Streetscape section*						
Transit	Transit type and amenities, informal places to catch transit	0–10	14	0.788 ICC	2.83 (2.70)	2.48 (2.39)
Traffic calming	Number of signs, circles, speed tables, speed humps, curb extensions in segment; recoded: 0 = 0, 1 = 1, 2 = 2, 3 = 3, 4 = 4, 5 + = 5	0–5	1	0.597 ICC	2.47 (1.98)	1.90 (1.78)
Street Amenities	Presence of trash bins, benches, bicycle racks, bicycle lockers/compounds, kiosks/information booths, hawkers/shops/carts.	0–5	6	0.891 ICC	1.45 (1.42)	1.33 (1.42)
Overall Streetscape Positive	Transit, traffic calming, street amenities	0–18	21	0.742 ICC	6.95 (4.45)	5.90 (4.00)
*Aesthetics and Social section*						
Overall Aesthetics and Social Positive	Hardscape, water, softscape, landscaping	0–4	4	0.612 ICC	1.03 (0.73)	1.09 (0.83)
Overall Aesthetics and Social Negative	Buildings not maintained, graffiti, litter, dog fouling, extent of disorder, highway nearby	0–6	6	0.738 ICC	2.74 (1.63)	2.36 (1.50)
Overall Aesthetics and Social Score	Positive + Negative	− 6–3	10	0.736 ICC	-1.71 (1.92)	-1.27 (1.98)

*SD* standard deviation

Segment and crossing subscale reliability and descriptive analyses are shown in Tables 3 and 4, respectively. The majority of subscales had ICCs higher than 0.80 (i.e., excellent reliability), and almost all subscales showed good reliability with ICCs higher than 0.60. Only two single item indicators (informal path or shortcut positive, and hawkers/shops positive) had low Kappa and ICC values due to insufficient variability, but those items had inter-rater agreements from 93.3–95.7%. The overall segment score had an ICC value of 0.929, and the overall crossing score had an ICC of 0.885.

**Table 3:** MAPS-Global Segment section reliability scores (n = 1370) between on-street and online raters

Subscale	Content	Range of scores	Number of items	Inter-Rater Agreement Kappa/ICC	Rater’s mean (SD)	
					On-street	Online
Building Height and Setbacks Positive	Setbacks/building height	1–13	3	0.897 ICC	5.73 (3.48)	5.48 (3.39)
Sidewalk Qualities Positive	Sidewalk presence and width	0–6	2	0.856 ICC	4.79 (1.70)	4.83 (1.56)
Buffers Positive	Parking and buffer	0–5	2	0.848 ICC	3.42 (1.72)	3.50 (1.70)
Bicycle Infrastructure Positive	Bike lane presence, quality, signage	0–5	3	0.856 ICC	0.32 (1.04)	0.38 (1.16)
Building Aesthetics and Design Positive	Windows, trichotomized	0–2	1	0.617 ICC	1.63 (0.62)	1.60 (0.61)
Shade Positive	Number of trees, percent shade from trees and other coverage	0–6	3	0.855 ICC	1.74 (1.56)	1.73 (1.50)
Pedestrian Infrastructure Positive	Mid-segment crossing, pedestrian bridge, covered place to walk, street lights	0–5	4	0.616 ICC	1.08 (0.75)	0.93 (0.74)
Informal Path or Shortcut Positive	Informal path connecting to something else, Yes or No	0–1	1	0.538 K	0.09 (0.28)	0.07 (0.26)
Hawkers/Shops Positive	Hawkers/shops on sidewalk or pedestrian zone	0–3	1	0.194 ICC	0.02 (0.13)	0.06 (0.31)
Building Height: Road Width and Setback Ratio Positive	Smallest and largest setbacks, building height and road width	0–3	5	0.688 ICC	1.10 (1.06)	0.93 (1.14)
Overall Segments Positive	Sum of positive segment subscales	3–34	25	0.927 ICC	20.19 (6.97)	19.75 (7.08)
Overall Segments Negative	Sidewalk not continuous, trip hazards, obstructions, cars blocking walkway, slope, fences, driveways	0–13	7	0.852 ICC	3.42 (3.41)	3.41 (3.18)
Overall Segments Score	Positive-Negative	− 8-34	32	0.929 ICC	16.74 (9.60)	16.33 (9.48)

*SD* standard deviation

**Table 4:** MAPS-Global Crossing section reliability scores between on-street and online raters

Subscale	Content	Range of scores	Number of items	n	Inter-Rater Agreement Kappa/ICC	Rater’s mean (SD)	
						On-street	Online
Crosswalk Amenities Positive	Crossing aids, marked crosswalk, high visible striping, different material, curb extension, raised crosswalk, refuge islands	0–4	7	799	0.907 ICC	0.93 (1.14)	0.92 (1.11)
Curb Quality Positive	Curb presence, curbs lined up, tactile paving	0–6	3	782	0.751 ICC	4.34 (1.65)	4.16 (1.75)
Intersection Control and Signage Positive	Yield signs, stop signs, traffic signal, traffic circle, walk signals, push buttons, countdown signal	0–4	7	799	0.888 ICC	1.01 (1.03)	0.97 (1.09)
Bicycle Positive	Waiting area, bike lane crossing the crossing, bike signal	0–2	3	797	0.817 ICC	0.08 (0.34)	0.07 (0.33)
Pedestrian Overpass Positive	Crossing on pedestrian overpass, bridge. Yes or No	0–1	1	799	99.4^a^	0.00 (0.04)	0.01 (0.07)
Overall Crossings Positive	Positive crossing (sum of all above)	0–15	21	780	0.895 ICC	6.39 (3.26)	6.15 (3.42)
Road Width Negative	Distance of crossing leg, including all traffic lanes	0–2	1	799	0.921 ICC	1.98 (1.14)	1.96 (1.10)
Overall Crossings Score	Positive Crossing – Road Width Negative	− 1–15	22	777	0.885 ICC	6.27 (3.09)	6.03 (3.25)

*SD* standard deviation

^a^Too rare to calculate Kappa, reporting percent agreement

Finally, Table 5 shows MAPS-Global grand scores and conceptual scale reliability results. Pedestrian infrastructure, pedestrian design, and bike facilities scores showed good to excellent reliability, with ICC values higher than 0.87. The MAPS-Global overall grand score had similar mean values for the on-street and online raters and demonstrated good to excellent reliability (ICC: 0.861).

**Table 5:** MAPS-Global Grand Scores and conceptual scale reliability (n = 331) between on-street and online raters

Item	Content	Range of scores	Number of items	Inter-Rater Agreement Kappa/ICC	Rater’s mean (SD)	
					On-street	Online
Grand Score Overall	(Overall DLU Positive + Overall Streetscape Positive + Overall Aesthetics /Social Positive + Overall Segments Positive + Overall Crossings Positive) – (Overall DLU Negative + Overall Aesthetics/Social Negative + Overall Segments Negative + Road Width Negative)	− 0.17–70	116	0.861 ICC	19.28 (15.42)	18.49 (12.89)
Pedestrian Infrastructure	Trail, pedestrian zone, sidewalk presence and width, buffer, shortcut, mid-segment crossing, pedestrian bridge, air-conditioned place to walk, low lights, overpass, crosswalk, refuge island	0-16.08	13	0.876 ICC	7.44 (3.00)	7.62 (3.05)
Pedestrian Design	Open-air market, trash cans, benches, kiosks, hawkers and shops, setback, visibility, pedestrian walk signals, push buttons, countdown signals, ramps, crossing aids	1-18.47	14	0.885 ICC	9.51 (4.21)	9.14 (4.28)
Bike Facilities	Bike racks, docking stations, lockers, bike lane, bike lane quality, signs, bike signal, bike box, bike lane crossing the crossing	0-7.67	9	0.925 ICC	0.77 (1.37)	0.83 (1.38)

*DLU* destination and land use,* SD* standard deviation

## Discussion

The present study in five diverse countries examined the reliability between on-street and online observations conducted by different raters using the MAPS-Global tool that was designed for international use. Results showed good to excellent agreement between on-street and online audits for most of the summary scores analyzed. Only a few subscales had Kappa or ICC values < 0.70 (23.3 %), with aesthetic and social environment variables having the lowest overall reliability values, though still in the “good to excellent” category. Present findings of high reliability of different observers across different data collection methods were very similar to a previous report of reliability of MAPS-Global across two independent observers using the on-street method [17]. Present results indicate that MAPS-Global can be used internationally with either the on-street or online method, if online imagery data are available and sufficiently recent. Therefore, the present study adds international data supporting acceptable to high reliability across on-street and online observations.

There is no consensus on the time efficiency when comparing on-street and online environment audits, not including travel time. Studies have reported online time savings [8, 10, 30], no differences [9] or even longer time to complete online audits [7]. This lack of consensus is also present across countries within our study (see Table 1). These differences could depend on such issues as the complexity of the environment, characteristics of the assessment tool, or even differences in computer speed. However, online assessments eliminate travel time and costs [9, 10]. Remote audits also address safety problems associated with dangerous neighborhoods [9] and allow researchers to conduct assessments across multiple sites or vast areas [10]. In general, authors appear to agree that Google Earth and Google Street View can be efficient tools for collecting data on micro-scale neighborhood characteristics [9].

However, online methods present limitations that should be considered. Although coverage is increasing rapidly, imagery is not available in many countries, on some streets, or in rural areas [7, 14]. Many of these gaps should be addressed over time, but gaps are likely to remain in the lowest income countries and in some countries that prohibit or greatly restrict image-gathering programs such as Google Street View. Limitations of the online method include the time difference between collection of the imagery and its online observation. A related limitation of the present study was lack of documentation of the date of the imagery and interval between imagery collection and observation. Some characteristics can be difficult to view due to the camera’s perspective, resolution, or parked or moving vehicles that could block the view of the sidewalks and buildings [7, 9, 10, 14]. Camera views of tall buildings also are restricted. These limitations might explain lower reliability results for aesthetic and social environment variables in the present study and in others [16]. However, these lower reliability results might also be explained by the transitory and subjective nature of these characteristics [31]. Temporal variability of Google Earth and Google Street View images and acquisition dates across locations should be taken into account when auditing multiple sites [7–9, 13].

Considering good inter-rater reliability and advantages of online audits, we conclude the MAPS-Global instrument can be used both on-street and online to analyze the micro-scale environment characteristics across diverse countries. The present findings also provide initial evidence to justify combining observations from both data collection methods in the same study due to good overall comparability. Next steps are to evaluate MAPS-Global in more countries, especially low-income countries, identify characteristics of the built environment that may moderate the reliability and validity of online audits (e.g., density), and assess construct validity in relation to physical activity and other outcomes. Further studies with larger samples are needed to examine whether there are differences across countries in reliability across observation methods. It would also be useful to evaluate whether it makes a difference if the rater is familiar with the country and language being observed, as online assessments from a central location could provide more efficient and standardized data collection for international studies.

MAPS-Global has been shown to have strong inter-observer agreement with in-person auditing [17], and present results showed acceptable agreement between in-person and online auditing in diverse countries. These results provide reassurance about the international applicability of MAPS-Global and its psychometric qualities. MAPS-Global data have been collected for a subsample of routes beginning at residences of a subset of participants in IPEN-Adolescent in eight countries [26]. These data can now be analyzed to address important questions related to health geography. Streetscape scores can be compared across diverse countries to understand the range and distribution of pedestrian- and bicycle-supportive environments. Differences in streetscape quality across lower- and higher-SES area can be examined. Central to the aims of IPEN-Adolescent, the relation of streetscape quality to adolescents’ physical activity patterns and weight status can be studied, and differences in associations across countries can be explored. We encourage other investigators to use MAPS-Global to answer a variety of important questions related to health and geography. MAPS-Global data can be used to develop evidence-based built environment recommendations for policies and practices that are either tailored to particular locales or applicable internationally.

## Research highlights

- The MAPS-Global streetscape audit tool was evaluated for reliability in 5 countries.
- The tool showed good-to-excellent reliability between on-street and online audits.
- MAPS-Global could be used both on-street and online internationally.

## Data Availability

The datasets used and analysed during the current study are available from the corresponding author on reasonable request.

## Abbreviations

MAPS: Microscale Audit of Pedestrian Streetscapes
ICC: Intraclass Correlation Coefficients
PA: Physical activity
IPEN: International Physical Activity and the Environment Network
SES: Socio-economic status
GIS: Geographical information system
SD: Standard deviation
